# Large anthropogenic impacts on a charismatic small carnivore: Insights from distribution surveys of red panda *Ailurus fulgens* in Nepal

**DOI:** 10.1371/journal.pone.0180978

**Published:** 2017-07-14

**Authors:** Saroj Panthi, Gopal Khanal, Krishna Prasad Acharya, Achyut Aryal, Arjun Srivathsa

**Affiliations:** 1 Department of Forests, Ministry of Forest and Soil Conservation, Kathmandu, Nepal; 2 Post-Graduate Programme in Wildlife Biology and Conservation, Wildlife Conservation Society, India Program, National Centre for Biological Sciences (Tata Institute of Fundamental Research), Bangalore, India; 3 Centre for Ecological Studies, Lalitpur, Nepal; 4 Charles Perkins Centre, School of Life and Environmental Sciences, Faculty of Science, The University of Sydney, Sydney, Australia; 5 Department of Forest and Resource Management, Toi Ohomai Institute of Technology, Rotorua, New Zealand; 6 Faculty of Science and Technology, Federation University Australia, Gippsland Campus, Churchill, Victoria, Australia; 7 Human-Wildlife Interaction Research Group, Institute of Natural and Mathematical Sciences, Massey University, Auckland, New Zealand; 8 School of Natural Resources and Environment, University of Florida, Gainesville, Florida, United States of America; 9 Department of Wildlife Ecology and Conservation, University of Florida, Gainesville, Florida, United States of America; University of South Carolina, UNITED STATES

## Abstract

Protected areas are key to preserving biodiversity and maintaining ecosystem services. However, their ability to ensure long-term survival of threatened andendangered species varies across countries, regions and landscapes. Distribution surveys can beparticularly important for assessing the value of protected areas, and gauging their efficacy incatering to species-specific requirements. We assessed the conservation value of one such reserve for a charismatic yet globally endangered species, the red panda *Ailurus fulgens*,in the light of on-going land-use transformation in Nepal. We conducted field surveys forindirect signs of red pandas along forest trails in 25-km^2^ sampling grid cells (n = 54) of Dhorpatan Hunting Reserve, and confronted a set of ecological hypotheses to the data using hierarchical occupancy models. We estimated overall occupancy at Ψ(SE) = 0.41 (0.007), with relatively high site-level detectability [*p* = 0.93 (SE = 0.001)]. Our results show that despitebeing a subsistence form of small-scale resource use, extraction of bamboo and livestock grazing negatively affected panda occurrence, albeit at different intensities. The amount of bamboo cover,rather than the overall proportion of forest cover, had greater influence on the panda occurrence. Despite availability of bamboo cover, areas with bamboo extraction and anthropogenic disturbances were less likely to be occupied by pandas. Together, these results suggest that long-term persistence of red pandas in this reserve and elsewhere across the species’ range will require preventing commercial extractionof bamboo, coupled with case-specific regulation of anthropogenic exploitation of red panda habitats.

## Introduction

Protected areas (PAs) have been crucial for biodiversity conservation efforts worldwide [1]. Covering about 15% of the global land area, PAs serve as the best defense against current biodiversity loss [2]. The extent and intensity of protection that PAs offer, however, vary across countries, regions and landscapes [3,4]. Some species have benefited from inviolate protected areas (e.g., tigers in India and Nepal: [5]), while others have thrived with reoccurring conflicts with humans [6], sustainable harvest of species and relatively benign human-wildlife interactions (e.g., sustainable trophy hunting of lion in Africa: [7–9]. Evaluating the mode of protection and its efficacy based on species- or system-specific requirements is important, yet seldom assessed in conservation studies.

Distribution surveys are important for making ecological inferences on species presence, their habitat and ecological requirements [10,11]. Large-scale distribution surveys can inform us on species ranges, meta-population structures and landscape-level attributes facilitating species persistence [12–14]. At smaller scales, within PAs, it allows for making inferences on habitat preferences, local threats, and responses to management interventions [15]. Consequently, insights from distribution surveys can be critical for making informed management decisions and conservation policy change [16].

Studies of mammals are generally biased towards large-sized species, in biodiversity rich regions [17,18]. Small carnivores, despite playing a disproportionately important role in the ecosystem [18], are among the least studied mammals, and scarce information exists on small carnivores from the Himalayas in particular [19,20]. The red panda *Ailurus fulgens* typifies this issue; despite being a charismatic small carnivore, there is serious dearth of quantitative assessments on its population and distribution status.

The red panda is found in moist and dry temperate forests from Nepal in the West through China, India, Bhutan and Myanmar in the East. Current estimates suggest that global potential red panda habitat cover 47,000 km^2^ [21]. About 10,000 mature red pandas are thought to remain in the wild, although reliable estimates of their abundance are not available [22]. They are difficult to detect, track, observe and study due to their elusive nature, arboreal habits, and occurrence in remote and inaccessible areas [23–25]. Despite being categorized in the Endangered category of the IUCN Red List, and widespread conservation efforts over the last decade [22], the species continues to face multiple threats, including loss/fragmentation of bamboo habitats due to livestock grazing and fodder collection, commercial logging, disease, and poaching for its pelts [25–28].

Previous studies of red panda ecology in Nepal Himalayas, Northeast India, and Bhutan largely focused on assessing their habitat and diet preferences [29–32]. One study by Kandel et al [21] generated a predictive map of the species’ geographic range limits. Unfortunately, these studies do not provide reliable information on the distribution status of red panda in the area because they ignore sampling biases arising from imperfect detection in field surveys. Ignoring imperfect detection can seriously underestimate the true distribution status of a species [33–35] and provide inadequate information on the relationships between species and ecological/anthropogenic attributes of its habitat [36].

In this study, we used hierarchical models that allow simultaneous estimation of occupancy (Ψ) while accounting for the observation process (detectability, *p*) to examine distribution patterns of the red panda in Dhorpatan Hunting Reserve, Nepal. We also investigated the influence of key ecological and anthropogenic factors on its occurrence. We predicted *a priori* that forest cover and bamboo presence would positively influence occupancy patterns, whereas human disturbance associated with livestock grazing, and bamboo and wood extraction would a have negative influence. Based on our findings, we identify key threats to persistence of pandas in this reserve, and, generate information on specific areas within the reserve that require management focus.

## Methods and materials

### Study area

We received the research permit by Department of National Parks and Wildlife Conservation (DNPWC) to conduct this study in Dhorpatan Hunting Reserve (23°30'N-28°50'N, 82°50'E-83°15'E). This reserve is among the most important areas identified for red panda conservation in Nepal [30,37]. It is the only hunting reserve in Nepal, which is famous for trophy hunting of ‘bharal’ or blue sheep (*Pseudois nayaur*) and Himalayan tahr (*Hemitragus jemlahicus)* [38]. Other mammals recorded in this reserve include Asiatic black bear (*Ursus tibetanus*), barking deer (*Munticus muntjak*), goral (*Naemorhedus goral*), Himalayan tahr (*Hemitragus jemlahicus*), leopard (*Panthera pardus*), rhesus macaque(*Macaca mulatta*), *Himalayan serow* (*Capricornis thar*), wild pig (*Sus scorfa*) and wolf (*Canis lupus)* [30,39]. Established in 1983, it covers an area of 1,325 km^2^. The elevation ranges from2,000 m to7,246 m above sea level. Temperate broad-leaved, mixed deciduous, and temperate evergreen are major forests types here, which include mixed patches of fir (*Abies spectibilis*), blue pine (*Pinus wallichina*), birch (*Betula utilis*),rhododendron (*Rhododendron spp*.), hemlock (*Tsuga dumosa*), oak (*Quercus semecarpifolia*),juniper (*Juniperus indica*) and spruce (*Picea smithiana*). *Arundinaria* spp.(bamboo) formsan under-story cover in these forests.

The reserve is divided into seven administrative blocks, namely Sundaha (145 km^2^), Seng (138 km^2^), Dogadi (199 km^2^), Ghustung (201 km^2^), Fagune (327 km^2^), Barse (167 km^2^) and Surtibang (148 km^2^). The reserve also has substantial human presence. Local communities extract bamboo mainly as fodder for livestock. Bamboo extracted from the reserve is also used to produce baskets and sold in local markets. Livestock grazing and fodder collection by local communities are widespread across the area, and these activities have high potential to affect local abundance and distribution of red pandas [28]. The effects of widespread resource use and extraction activities on abundance and distribution of red pandas are not fully understood.

### Study design

**Fig 1:**
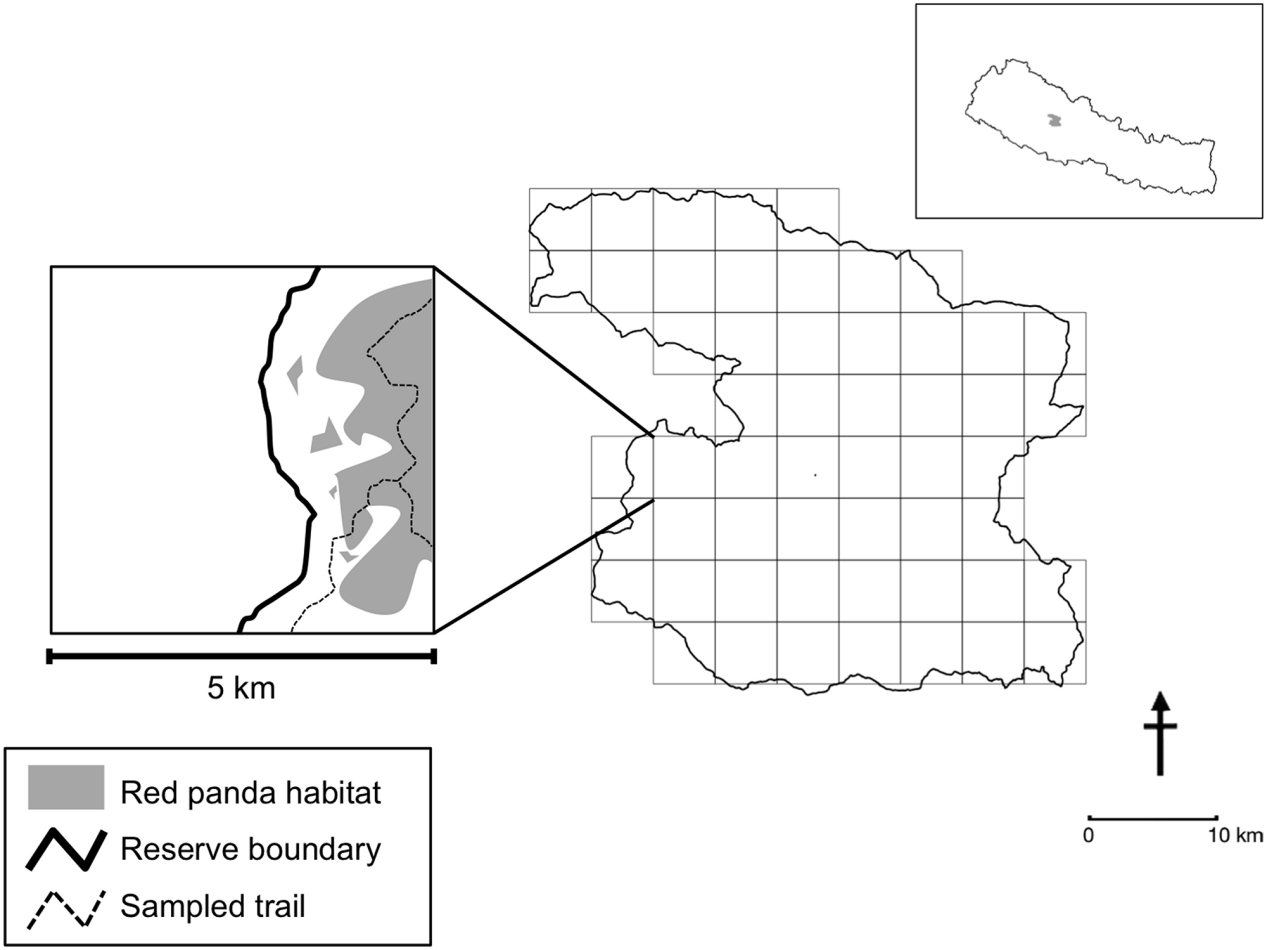
Map of Dhorpatan Hunting Reserve, with grid overlay on the study region and schematic of field survey design. Inset: Geographic location of the study area in Nepal.

Our key parameter of interest was occupancy (the proportion of area occupied by red pandas). We divided the study area into spatial sub-units with an array of square-shaped grid cells measuring 5 X 5 km^2^ each. To estimate true occupancy, the size of sampling unit should be larger than the home range size of the species [33]. The size of our grid-cells (25 km^2^) was therefore larger than the documented home range size of red pandas (2.2±1.21 km^2^; [40]). We used QGIS ver. 2.18.0 [41] to overlay geographical grids cells on the land cover matrix of the study area (Fig 1). We treated 54 such grid cells as independent sites in our analysis. We divided each site into 2.5 X 2.5 km^2^sub-sites, and randomly chose one sub-site as a starting point of the survey prior to fieldwork, so as to not induce systematic sampling bias. In each site, we laid a minimum of 4 to a maximum of 10 1-km long transects, with at least 300 m gap between consecutive transects to ensure spatial independence between adjacent transects. We further divided each 1-km long transect into four 250 m segments to record detection (1s) and non-detection (0s) of red panda signs, and to measure covariate values predicted to influence occupancy and detection probabilities. Transects were laid along human trails, forest roads, and microhabitats that are likely to be used by red pandas (see Fig 1) and field surveys were conducted from June to July 2013. Given the tough terrains and difficulties in accessing certain regions of our study area, we chose to use spatial replication instead of temporal replication [42,43]. Each 1-kilometer segment sampled was treated as a spatial replicate for the corresponding site. We used only fresh pellets as detections in our analysis, discarding detections of old fecal pellets to adhere to the closure assumption in occupancy studies [44].

### Ecological and anthropogenic variables

We selected seven plausible covariates relevant to ecology of the species and anthropogenic interventions in the study area (Table 1). Multiple studies have shown that red pandas prefer thick bamboo under-story habitats [30]. *Arundinaria* bamboo is known to contribute about 81.7% of red panda diet in this reserve [30]. The covariate *bamboo* in this study reflects the proxy for availability of bamboo habitat, mostly comprised of *Arundinaria* spp. in each site (calculated for each site as a ratio of the number of segments with bamboo and total number of sampled segments in a site). Previous studies in this reserve have documented red panda presence mostly in lower elevation areas, below 4000 m [30]. Recognizing that red pandas have a narrow range of preference for elevation (*c*. 2600-3600m; [45,46]), we measured average elevation in each site as a potential predictor of panda occurrence. We also expected that amount of forest cover in each site would influence occupancy [47]. We calculated the proportion of the forest area per grid cell using QGIS ver. 2.18.4 by processing remotely sensed satellite images obtained from Landsat data (https://earthexplorer.usgs.gov/). In quantifying human-induced disturbance, we considered three indices that likely affected red panda occupancy: (1) livestock grazing, (2) wood extraction, and (3) bamboo extraction. These indices were calculated as ratios, similar to the index for bamboo availability described above. In addition to these site-based covariates, we also used number of replicates per site as a covariate for site-level detectability, since sampling effort varied across sites (see Table 1 for details on covariate information).

**Table 1:** Description of ecological and anthropogenic covariates and their predicted influence (direction) on parameters of interest: Site-level occupancy probability (ψ), and detection probability (*p*); *a priori* predictions about their influence on probability of red panda occupancy are also described. The relationship between the parameter of interest and the covariate is assumed to be linear (on the logit scale) unless specified otherwise.

Covariate	Source and description	Expected influence on occupancy and detection probability
***forest (for)***	Proportion of forest area: Amount of forest habitat within each sampling grid-measured by Landsat data (https://earthexplorer.usgs.gov/).	Positive effect on both ψand *p* as increase in forest cover is expected to be correlated with higher food resources
***bamboo (bam)***	Index of bamboo forage availability: Proportion of 1-km spatial replicates within a site with presence of *Arundinaria* spp. (bamboo grass)	Positive effect on both ψand *p*. Presence of bamboo indicates higher food availability for red panda, and higher local abundance of pandas would enable higher detectability
***elevation (elev)***	Calculated using ASTER Ver. 2. Global Digital Elevation Model in QGIS	Study area ranges between *c*.3000 and 7000 m elevation. We expected a negative relationship since red pandas prefer lower elevations
***bamboo extraction (bex)***	Proportion of 1-km spatial replicates in a site with signs of bamboo lopping and extraction	Negative effect on both ψand *p* reduced forage availability, and indirectly through correlated anthropogenic disturbances
***livestock grazing (lvs)***	Proportion of 1-km spatial replicates in a site with signs of livestock grazing.	Negative effect on both ψand *p* due to reduced forage availability and indirectly through trampling of bamboo stands, and correlated anthropogenic disturbances
***wood extraction (wex)***	Proportion of 1-km spatial replicates in a site with signs of wood extraction	Negative effect on both ψand *p* directly by reducing the availability of habitat and indirectly affecting nesting, shelter and resting locations and feeding habits
***effort (eff)***	Number of surveyed replicates in each site	Positive effect on *p* since higher effort in a site would increase probability of detecting signs

### Data analysis

A detection/non-detection matrix of red panda occurrence was constructed with ‘1’ as detection and ‘0’ as non-detection. We z-standardized all predictor variables (Table 1) so that the model coefficients could be directly interpreted as effect sizes. We also assessed cross-correlations between the predictor variables using Pearson’s coefficients. We constructed covariate combinations such that correlated predictors (Pearson’s |*r|*> 0.7) did not appear in the same model [48]. All analyses were performed on program PRESENCE *v*. *11*.*5* [49] using the standard single season occupancy model described by MacKenzie et al. [33]. The covariate models were compared and ranked using an information theoretic approach, relying on Akaike Information Criterion (AIC; [50]) for testing relative model fits.

We adopted a two-step approach to obtain our parameter estimates. In the first step, we defined a general structure for occupancy Ψ (*bamboo+forest+livestock*) and modeled detection probability (*p*), either as an intercept-only model, i.e., *p*(.), or as a function of individual covariates and their combinations. We expected that red panda sign detection probability would be high in sites with less human disturbance, as human presence such as livestock grazing and intensive bamboo extraction can reduce the visibility of the signs. In addition, we hypothesized that sampling grids with presence of *Arundinaria* spp. (bamboo) and greater proportion of forest area would have higher probability of detecting red panda signs, presumably due to higher food resources, and consequently higher local abundances of red pandas.

In the second step, we used the best-fit detection probability model from the previous step and modeled the occupancy probability (Ψ) as a function of covariate combinations (Table 2). We constructed a set of 23 a *priori* candidate models, each representing a different ecological hypothesis. These models included either single or additive effects of two or more covariates (on the logit scale). Models with ΔAIC of <2 were considered to be strongly supported by the data. We used the estimated β-coefficients to assess the strength of association of each covariate with occupancy probability. Model fit was assessed for over-dispersion by running bootstrap goodness-of fit tests for the best-fit occupancy model (n = 1000, bootstrap samples). Values of c-hat >1 indicate that there is more variation in the observed data [51]. Values of c-hat <1 indicate less variation than expected and do not pose a problem [50].

**Table 2:** Summary of model comparisons showing effects of covariates on detection probability (Step 1) and occupancy (Step 2) of red panda *Ailurus fulgens* (n = 54 sites). Akaike’s information criterion (AIC), change in AIC (ΔAIC), Akaike weights, model likelihood, number of parameters (K), and deviance (-2log-likelihood). Covariates used are: *bam*, proportion of bamboo availability; *for*, proportion of forest area; *lvs*, livestock grazing intensity; *bex*, intensity of bamboo extraction; *elv*, average elevation in a site; *eff*, number of replicates surveyed in each site.

Model	AIC	ΔAIC	AIC weight	Model likelihood	K	Deviance
**Step 1**						
*ψ (bam+for+lvs)*,*p(bex+bam)*	327.43	0	0.1461	1	7	313.43
*ψ (bam+for+lvs)*,*p(bex+bam+for)*	327.73	0.3	0.1258	0.8607	8	311.73
*ψ (bam+for+lvs)*,*p(bex+for)*	328.19	0.76	0.0999	0.6839	7	314.19
*ψ (bam+for+lvs)*,*p(bex+bam+lvs)*	328.57	1.14	0.0826	0.5655	8	312.57
*ψ (bam+for+lvs)*,*p(bex)*	328.78	1.35	0.0744	0.5092	6	316.78
*ψ (bam+for+lvs)*,*p(for)*	328.84	1.41	0.0722	0.4941	6	316.84
*ψ (bam+for+lvs)*,*p(bex+bam+eff)*	329.33	1.9	0.0565	0.3867	8	313.33
***Step 2***						
*ψ (elv+bam+bex)*,*p(bex+bam+eff)*	308.34	0	0.1932	1	8	292.34
*ψ (elv+bam+bex)*,*p(bex+bam)*	308.49	0.15	0.1792	0.9277	7	294.49
*ψ (bam+bex)*,*p(bex+bam+eff)*	309.98	1.64	0.0851	0.4404	7	295.98
*ψ (elv+bam+bex+lvs)*,*p(bex+bam+eff)*	310.12	1.78	0.0793	0.4107	9	292.12
*ψ (for+bam+bex+lvs)*,*p(bex+bam)*	312.54	4.2	0.0237	0.1225	8	296.54
*ψ (elv+bex)*,*p(bex+bam)*	316.42	8.08	0.0034	0.0176	6	304.42

## Results

We surveyed 54 sites, covering an area of 1149.95 km^2^. A total of 425 km walk effort was invested, with a mean of 7.8 km (range 4–10 km) across the sites. At least one sign was detected in 21 sites; this resulted in naïve occupancy of 0.39. Cross-correlation tests between pairs of covariates indicated that forest cover was correlated with elevation (Pearson’s correlation *r* = -076). We therefore did not use these two covariates together in any of the model combinations.

The top-ranked model for detectability of red panda signs showed that the additive effect of bamboo availability and bamboo extraction best fit the data (Table 2). The probability of detecting red pandas increased with bamboo availability (β_bam_ = 0.36 ± SE 0.19) and decreased with bamboo extraction (β_bex_ = -0.92±SE0.28). We used these two covariates for detectability in subsequent analyses to model Ψ. Among the set of candidate occupancy models, the model with Ψ as a function of bamboo availability (β_bam_ = 3.73±SE 1.61), elevation (β_elv_ = -1.47±SE 0.86) and bamboo extraction (β_bex_ = -3.97±SE 1.58) received most support from the data (Table 2). Although it did not feature in the top-ranked model from the first step, we still explored the influence of unequal survey effort (*eff*) on detectability in these models and derived final estimates of occupancy and detectability from the model with the best relative fit. The goodness-of-fit test for the top-ranked occupancy model indicated no over dispersion of observed data (c-hat = 1.05, p >0.05).

**Fig 2:**
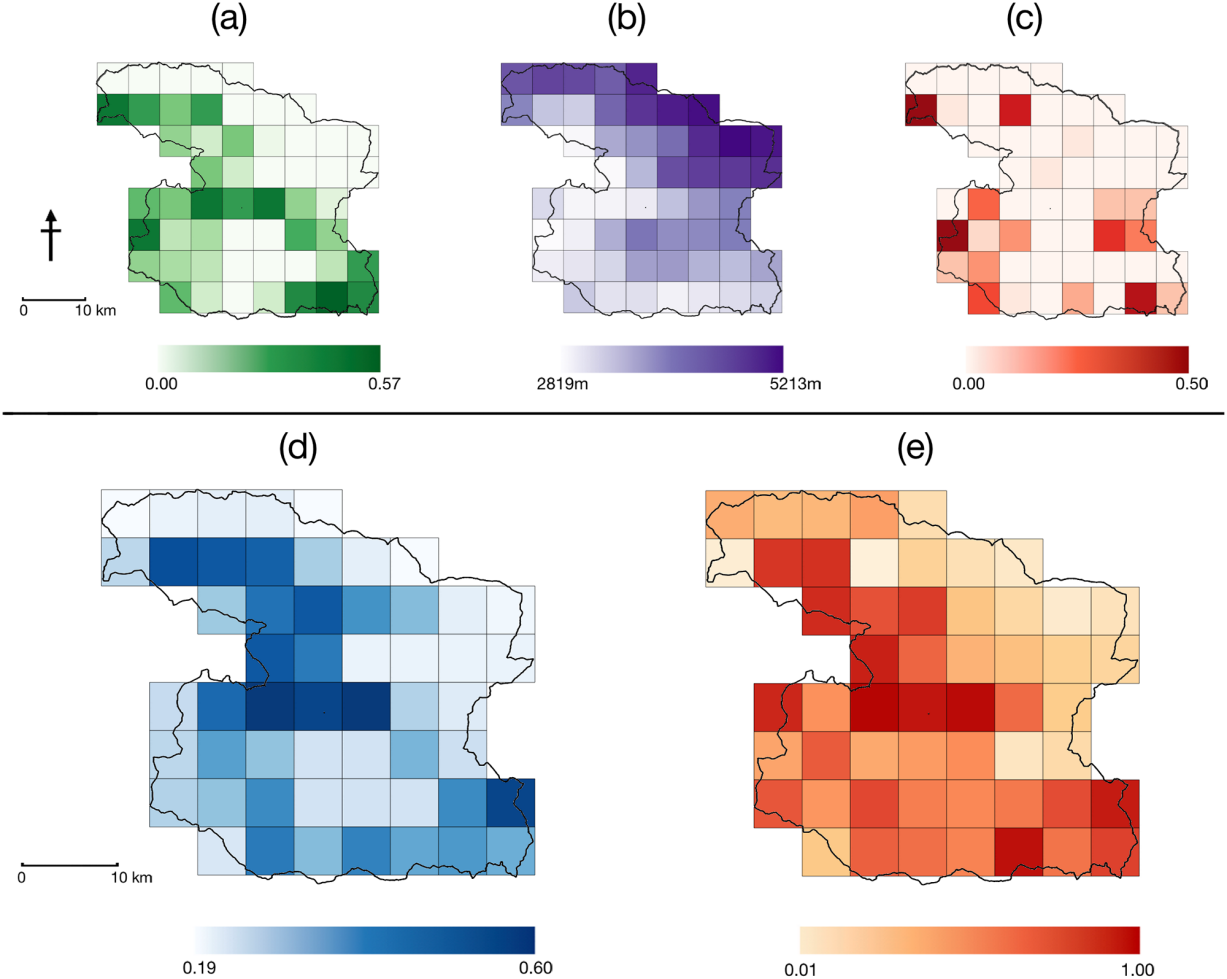
Spatial patterns of key covariates (a) bamboo availability, (b) elevation, (c) bamboo extraction, and predicted probabilities of (d) detection of red panda signs and (e) red panda occupancy in Dhorpatan Hunting Reserve.

Since a single model did not fully explain the observed data, model specific β-coefficient estimates from the top nine models (with AIC weight >0.01) are provided in Table 3. Estimated β-coefficients from these nine models showed that (1) red panda presence was positively associated with presence of bamboo grass, as expected, (2) red pandas preferred lower elevations within the study area, and (3) they avoided areas with human disturbance, specifically areas prone to bamboo extraction. The 95% confidence intervals for β-coefficients of *bamboo and bamboo extraction* did not straddle zero, indicating that they have a significant effect on red panda occurrence. However, β-coefficients for livestock grazing, wood extraction and proportion of forest area had relatively lower reliability. Although elevation appeared in the top model, the associated standard error with the slope parameter was high, which resulted in marginal overlap with zero in its 95% confidence intervals (-3.16 to 0.21). Bamboo availability (cumulative AIC weight = 0.80 for candidate models) and bamboo extraction (cumulative AIC weight = 0.62) best explained the variability in site-specific occupancy probability (Table 3). Since all three top models in the candidate set (with ΔAIC = 1.64) were nested, we inferred overall occupancy estimates from the top model. Based on this model, the average detection probability (*p*) was 0.93 (SE = 0.001; calculated for each site as p* = 1-((1-*p*)^*k*^, where *k* = number of replicates) and ranged from 0.56 to 0.99. Overall probability of occupancy was 0.41 (SE = 0.07) and site-wise probabilities ranged from 0.005 to 1. The site-specific estimates of red panda occupancy are mapped in Fig 2.

**Table 3:** Summary of model-specific β-coefficient estimates and summed Akaike weights for covariates hypothesized to influence red panda occurrence in Dhorpatan Hunting Reserve, Nepal. Covariates:^:^ *bam*, proportion of bamboo availability; *for*, proportion of forest area; *lvs*, livestock grazing intensity; *bex*, intensity of bamboo extraction; *elv*, average elevation in a site; *eff*, number of replicates surveyed in each site.

Model	β_0_ (SE)	β_elev_(SE)	β_bam_ (SE)	β_bex_(SE)	β_for_(SE)	β_lvs_(SE)	AIC wt.
*ψ (elv+bam+bex)*,*p(bex+bam+eff)*	0.15 (0.86)	-1.47 (0.86)	3.73(1.61)	-3.97(1.57)	-	-	0.1932
*ψ (elv+bam+bex)*,*p(bex+bam)*	-0.21(1.15)	-1.47 (0.83)	3.01 (2.57)	-3.26 (2.52)	-	-	0.1792
*ψ (bam+bex)*,*p(bex+bam+eff)*	0.71 (0.86)	-	4.85(1.72)	-4.34 (1.68)	-	-	0.0851
*ψ (elv+bam+bex+lvs)*,*p(bex+bam+eff)*	0.18(0.84)	1.42(0.87)	3.84 (1.61)	-4.03 (1.55)	-	0.26(0.56)	0.0793
*ψ (elv+bam+bex+lvs)*,*p(bex+bam)*	-0.05 (0.10)	-1.47 (0.84)	3.42 (2.18)	-3.62 (2.10)	-	0.22 (0.58)	0.0707
*ψ (for+bam+bex)*,*p(bex+bam)*	-0.39 (0.58)	-	2.50 (1.04)	-2.46 (0.92)	0.88 (0.59)	-	0.063
*ψ (bam+bex)*,*p(bex+bam)*	0.36 (1.07)	-	0.35 (2.38)	-3.79 (2.29)	-	-	0.0568
*ψ (for+bam+bex)*,*p(bex+bam+eff)*	0.41 (0.88)	-	4.06 (1.78)	-3.88 (1.66)	0.62 (0.64)	-	0.0501
*ψ (for+bam+bex+lvs)*,*p(bex+bam)*	-0.38 (0.60)		2.57(1.41)	-2.50(0.99)	0.88(0.60)	0.10(0.51)	0.02
**Summed Akaike Weight**		**0.52**	**0.80**	**0.62**	**0.33**	**0.36**	

## Discussion

Reserve-level assessments of species’ habitat requirements and their responses to anthropogenic resource extraction patterns are critical to make informed decisions about conservation and management of threatened or endangered species [52]. In this study, we addressed ecological hypotheses relevant to evidence-based conservation and management of red pandas and their habitat in Dhorpatan Hunting Reserve, Nepal. Overall, our results show that red pandas occupied less than 50% of the study area, and were negatively affected by natural resource extraction currently prevalent in the reserve. There was high variation in site-specific occupancy probabilities within the reserve, suggesting that some of these areas might require considerable management focus.

### Red panda occurrence and habitat correlates

Bamboo availability, its extraction, and elevation were found to be the most important predictors of red panda occurrence. Observed variation in red panda occupancy probability in the study area was better explained by the presence of bamboo, than by the overall proportion of forest cover in the sampling grid. Red pandas generally inhabit dense bamboo thicket under-stories [24,30,45,53]. Being bamboo specialists, more than 80% of their diet consists of bamboo grass [29–32,54]. Previous studies in this region also have shown strong associations between red panda presence and habitats with understorey cover dominated by bamboo grass [26,53]. Red pandas occur in temperate forests ranging from deciduous broad leaved forests to conifer forests. But tree species in these habitats, like *Abies spectabilis*, *Rhoderndron arboreum*, *Acer* spp., *Betula* spp., and *Quercus* spp. contribute little to their diet, though they enhance habitat availability and connectivity between habitat patches [30,55]. The lack of any discernible relationship between red panda occurrence and proportion of forest area in our study could be because of the homogeneity in forest types across the sites (temperate mixed deciduous and evergreen forests). However, it is important to note that forest cover that includes species like *Abies* spp., oaks, and *Pinus* spp. have been increasingly eliminated from the region to meet timber demands of local communities. These tree species provide important resting and nesting cover for red pandas [45], and increased human extraction of timber could be detrimental to them.

The average elevation of the sampling units (sites) in our study ranged from 2820 m to about 5253m above sea level. Higher elevation areas in Nepal Himalayas are often associated with reduced forest cover, with dominance of mountain rangelands and alpine meadows [56]. Reduced probability of red panda occurrence in higher elevation in our study area probably reflects their inability to use such areas with scarce patches of preferred habitat. This is consistent with previous studies across the species’ range, which have shown that red panda generally avoided areas above 4000m elevation due to non-availability of preferred habitat [30,46,57,58].

### Anthropogenic threats and interactions with humans

Red pandas avoided areas with high levels of bamboo grass extraction. Local communities residing in the periphery of the reserve directly depend on bamboo as a source for livestock fodder. Bamboo is also used for making house roofs, scaffolding, container poles, and importantly toproduce baskets and other handicrafts, which are often sold commercially. The tender young shoots are also eaten as vegetables [59]. The Mountainous National Parks Regulation (1979) in Nepal allows for subsistence extraction of forest resources (including bamboo). Although collection of bamboo for commercial purposes is legally prohibited inside Dhorpatan, high dependency of local communities on forest resources for subsistence and poor law enforcement continues to remain a challenge. Local accounts with PA officials confirmed that the demand for these products has been growing over the years and will likely continue to increase (S. S. Thagunna, pers. comm.). It is therefore plausible that increased extraction of bamboo will induce higher habitat degradation through loss of forage (for pandas) and correlated human disturbance. Bamboo extraction, if not properly regulated, can be a major threat to persistence of red pandas in this reserve.

Despite being a protected area, around 47 human settlements (villages) exist inside Dhorpatan. Contrary to our expectation, we did not find a strong influence of livestock grazing and wood extraction on either detection or occupancy probabilities of red pandas. Several earlier assessments have, however, demonstrated negative influence of livestock grazing, and fodder and timber extraction on red panda presence [26,53,60,61]. Sharma et al. [60] found reduced site-use by red pandas in timber-extracted sites in Rara National Park, Nepal. Panthi et al. [30] showed that there was considerable overlap in resource-use by red pandas and livestock in Dhorpatan, with human disturbances and livestock grazing observed in 53% of red panda habitats in the reserve. Local knowledge and anecdotal evidence suggests that >3000 livestock heads exist inside the reserve area(B.K. Bhandari, pers. comm.). Livestock grazing not only reduces forage availability for red pandas, but their movement within the reserve compacts the soil and forms gullies, while also decimatingbamboo seedlings through trampling [26].

While our data did not show any detectable influence of wood extraction on panda occurrence, we note that fuel wood is still a dominant form of energy use in mid hills of Nepal, including Dhorpatan [62,63]. Small-scale urbanization in the region [64] has led to increased demand for timber for construction [65]. Any increase in logging and extraction of timber could be detrimental to red pandas, directly by reducing habitats and indirectly by severing connectivity among habitat patches.

### Spatial scale and study design limitations

Our field surveys were conducted in the months of April and May, the period when herders typically take their livestock to higher elevations above 4000 m (areas not preferred by red pandas) for grazing, as new flushes of vegetation and palatable grasses emerge after the first snow melt in late March. This seasonal pattern in spatial segregation of livestock grazing and red panda distribution during the study period could perhaps explain the lack of a significant relationship between the two. Assessments of their distribution across seasons [47] would therefore be essential to detect if livestock grazing directly affects red pandas in our study area.

Although our models did not reveal strong predictive power of wood extraction on red panda occupancy, we did record some forms of timber extraction, lopping of branches for fuel wood, and fallen logs and stumps in 29 out of 54 sites. While our study explicitly addressed imperfect detection of animal signs in the modeling process, we submit that the role of ecological and anthropogenic predictors we present could vary with spatial scale. For instance, our results did show a negative association of red panda with livestock grazing and wood extraction, but the effects were statistically indiscernible. It is likely that the two factors show stronger negative effects at finer spatial scales. Other habitat variables like water availability, forest type, and distance to human settlements could also affect panda occurrence, which we were unable to consider in this analysis. Most importantly, we did not incorporate the effect of poaching or poaching risk, although it is a potential threat to red pandas (few cases of poaching have been recorded in this reserve; two red panda pelts were confiscated from the livestock shed in Barse block in 2010 [66]).

### Conservation implications

Reliable information on abundance and distribution of species is central to understand their population status and inform management about measures to ensure their survival [67]. Our study is the first to present a robust estimate of red panda occupancy, and this could serve as a useful baseline for future monitoring of their ecology in Dhorpatan. The methods we use are cost-effective, and also provide more reliable estimates compared to most earlier studies, which use presence-only or presence-absence data to make inferences on species-habitat relationships [21,68].

We showed that anthropogenic impacts associated with bamboo extraction, livestock grazing and wood extraction negatively affected red panda occurrence, albeit at different intensities. Apart from being ideal red panda habitats, the temperate forestswith abundant under-story cover of bamboo grass in Nepal Himalayas(including Dhorpatan) are integral to the subsistence of local communities [69]. This dependency on red panda habitat presents significant challenges to its conservation, as increasing human population in the reserve frontier areas can escalate demands for bamboo and timber extraction for commercial purposes [70]. Other protected areas where red pandas occur, such as Langtang National Park, Rara National Park and Kanchenjunga Conservation Area, face similar pressures from livestock grazing and bamboo/wood extraction [31,47,71]. Regulation of bamboo extraction in areas of high panda occupancy could benefit the species in the short term. Alternative strategies could include promoting bamboo plantation in private lands and building local capacity to make efficient use of bamboo, thereby reducing the amount of produce required to meet subsistence needs. Long-term persistence of red pandas in this reserve and elsewhere across their geographic range will require preventing commercial exploitation of bamboo, coupled with spatio-temporal regulation inhuman use of red panda habitats.

## Supporting information

S1 File Site-wise detection history (detection/non-detection matrix) of red panda signs and corresponding ecological/anthropogeniccovariates used in the analysis. (XLSX)
